# Antidepressant sertraline increases thioflavin-S and Congo red deposition in APPswe/PSEN1dE9 transgenic mice

**DOI:** 10.3389/fphar.2023.1260838

**Published:** 2024-01-08

**Authors:** Ming-Hsuan Liao, Yen-Kuang Lin, Fong-Ying Gau, Chun-Che Tseng, Da-Chih Wu, Chu-Yuan Hsu, Kuo-Hsuan Chung, Rung-Chi Li, Chaur-Jong Hu, Chee Kin Then, Shing-Chuan Shen

**Affiliations:** ^1^ Graduate Institute of Medical Sciences, College of Medicine, Taipei Medical University, Taipei, Taiwan; ^2^ Graduate Institute of Athletics and Coaching Science, National Taiwan Sport University, Taoyuan, Taiwan; ^3^ School of Nursing, College of Nursing, Taipei Medical University, Taipei, Taiwan; ^4^ School of Pharmacy, College of Pharmacy, Taipei Medical University, Taipei, Taiwan; ^5^ Department of Psychiatry and Psychiatric Research Center, Taipei Medical University Hospital, Taipei, Taiwan; ^6^ Department of Psychiatry, School of Medicine, College of Medicine, Taipei Medical University, Taipei, Taiwan; ^7^ Division of Allergy and Immunology, University of Virginia, Charlottesville, VA, United States; ^8^ Department of Neurology, Shuang Ho Hospital, College of Medicine, Taipei Medical University, Taipei, Taiwan; ^9^ Graduate Institute of Clinical Medicine, College of Medicine, Taipei Medical University, Taipei, Taiwan; ^10^ MRC Oxford Institute for Radiation Oncology, Department of Oncology, University of Oxford, Oxford, United Kingdom; ^11^ Department of Radiation Oncology, Shuang Ho Hospital, Taipei Medical University, New Taipei City, Taiwan; ^12^ International Master/Ph.D. Program in Medicine, College of Medicine, Taipei Medical University, Taipei, Taiwan; ^13^ Department of Dermatology, School of Medicine, College of Medicine, Taipei Medical University, Taipei, Taiwan

**Keywords:** sertraline, paroxetine, SSRI, β-amyloid, Alzheimer’s disease, APP/PSEN1

## Abstract

**Introduction:** Depression is strongly associated with Alzheimer’s disease (AD). Antidepressants are commonly used in patients before and after their diagnosis of AD. To date, the relationship between antidepressants and AD remains unclear.

**Methods:** In our study, we administered sertraline or paroxetine to wild type (WT) and APPswe/PSEN1dE9 (APP/PSEN1) transgenic mouse models for up to 12 months. We quantified the drug concentrations using LC-MS/MS analysis and measured serum serotonin level using an ELISA assay. Additionally, we evaluated the amyloid burdens through thioflavin-S and Congo red stainings, and recognition memory using the novel object recognition test.

**Results:** Our findings revealed that mice treated with paroxetine exhibited a significantly higher level of weight gain compared to the control group and increased mortality in APP/PSEN1 mice. After 12 months of antidepressant treatment, the sertraline level was measured at 289.8 ng/g for cerebellum, while the paroxetine level was 792.9 ng/g for cerebellum. Sertraline significantly increased thioflavin-S and Congo red depositions, along with gliosis, in both isocortex and hippocampus of APP/PSEN1 mice compared to the control group. Both antidepressants also led to a decreased recognition index in APP/PSEN1 mice.

**Conclusion:** These findings suggest a potential role of sertraline in AD pathogenesis, emphasizing the need to reassess the use of these antidepressants in patients with AD.

## Introduction

Alzheimer’s disease (AD) is the most common cause of dementia, and is characterized clinically by a progressive and gradual decline of cognitive function. It is highly prevalent, incurable (Prince et al., 2013) and leads to huge social and economic burden around the world (Wimo et al., 2013). AD is characterized by reduced cholinergic transmission and neuronal death. Currently there is no cure for AD and the available treament, acetylcholinesterase inhibitors only provide some symptomatic relief (Mangialasche et al., 2010). The misfolded and aggregated proteins, β-amyloid (Aβ) peptide and hyperphosphorylated tau, have been postulated to be the cause of AD (Bloom, 2014). They have been used as biomarkers for diagnostic purposes (Braak and Braak, 1991; Thal et al., 2002) and therapeutic targets (van Dyck, 2018).

Depression is a common symptom which happen before or after the onset of AD and profoundly reduces the quality of life of AD patients. There are several possible hypotheses linking these two clinical entities: 1) Depression predisposes to AD, 2) depression presents as a symptom of AD, and 3) they are comorbidities which share similar pathogenesis (Saczynski et al., 2010; Byers and Yaffe, 2011; Li et al., 2011). As first-line treatment options for depression, the safety of antidepressants should be assessed in AD. The prevalence of antidepressant use has gradually increased around the world, especially the selective serotonin reuptake inhibitors (SSRIs) and serotonin-norepinephrine reuptake inhibitors (SNRI), including in United Kingdom (Mars et al., 2017), United States (Pratt et al., 2017), the Netherlands (Noordam et al., 2015) and Taiwan (Wu et al., 2012).

Mounting evidence suggests that antidepressant medication is associated with an increased risk of dementia (Wang et al., 2018; Chan et al., 2019). In the Taiwanese database with a million datasets, our study showed that antidepressants may increase the risk of dementia, independent of the presence of depression (Then et al., 2017a). Consistent with this result, Heath et al. showed that paroxetine is associated with higher risk of dementia in elderly population (Heath et al., 2018). Our *in vitro* study also revealed that sertraline and paroxetine, two SSRIs, induced astrocyte apoptosis by triggering calcium overload (Then et al., 2017b). Sertraline was seen to possibly alter brain structures, including the hippocampus and anterior cingulated, in depressed or non-depressed monkeys (Willard et al., 2015). Morever, sertraline is toxic and causes behavioral alterations in planarian which serves as an alternative model for the study of neurotoxicity (Hagstrom et al., 2015; Thume and Frizzo, 2017). However, several experimental studies using transgenic AD mouse model (Nelson et al., 2007) or investigating human participants (Cirrito et al., 2011) have been published, showing the potential of antidepressants in ameliorating β-amyloid pathology, while some indicate no significant effect (Severino et al., 2018).

Neuroinflammation, characterized by astrocytosis and microgliosis, is driven by amyloid pathology and also exacerbates the pathogenesis of AD (Selkoe, 2001; Hensley, 2010). Misfolded and aggregated proteins evoke an innate immune response via binding to pattern recognition receptors of micro- and astroglia, which is associated with disease progression (Heneka et al., 2015). Forst et al. also proposed that reactive astrocytes surround Aβ plaques and contribute to the overall amyloid burden in the brain (Frost and Li, 2017). These studies highlighted that glial cells amplify neuronal damage via enhancement of neuroinflammation. Therefore, controlling the proinflammatory process could be a therapeutic approach in AD (Bronzuoli et al., 2016).

Human population studies and *in vitro* studies showed that antidepressants, including sertraline and paroxetine, are associated with the risk of dementia or AD. The high prevalance of antidepressant medications in the world, coupled with a limited investigation of the consequences of chronic treatment with SSRIs, especially sertraline and paroxetine, highlights the importance to re-assess their safety in terms of AD. Therefore, we conducted an *in vivo* study for up to 12 months to investigate the impact of sertraline and paroxetine on thioflavin-S and Congo red deposition in AD transgenic (APP/PSEN1) mouse model.

## Materials and methods

### Animals

We obtained approval from the Taipei Medical University Institutional Animal Care and Use Committee. We used B6C3 hybrid background (C57BL/6 X C3H/HeN), double transgenic (Tg) APPswe/PSEN1dE9 (APP/PSEN1) mice (Jankowsky et al., 2001), and littermate wild type (WT) mice which were purchased from National Applied Research Laboratory, Taiwan (N = 66, n = 12 for each wild type group and n = 11 for each AD group). Mice were genotyped in-house with primers for APP (transgene F-AGGACTGACCACTCGACCAG, R-CGGGGGTCTAGTTCTGCAT; internal positive control F-CAAATGTTGCTTGTCTGGTG, R-GTCAGTCGAGTGCACAGTTT) and PSEN1 (transgene F-AATAAGAACGGCAGGAGCA, R-gCCATGAGGGCACTAATCAT; internal positive control F-CTAGGCCACAGAATTGAAAGATCT, R-GTAGGTGGAAATTCTAGCATCATCC). Only male mice were used in this study to avoid the confounding factor of mouse estrous cycle. All mice, regardless of their WT or APP/PSEN1 status and antidepressant treatment, were randomly allocated to cages. Between two and five mice were housed in each cage, depending on the supply conditions. Animals were fed with food and water *ad libitum* in 12 h light/dark cycle with constant temperature and humidity. We inspected the mice daily and recorded body weight weekly to assess their health. During euthanisation, an overdose of anaesthetic agents was used at study termination.

### Treatments of sertraline and paroxetine

Sertraline (CenZoft concentrate solution 20 mg/mL, Center) or paroxetine (Seroxat 15 mg, GSK) was administered in drinking water initially at a dose of 25 mg/kg/day and 10 mg/kg/day respectively. Treatment was initiated at 3 months of age, and was continued for a duration of 12 months. However, we observed unexpected sudden mortalities in APP/PSEN1 transgenic mice treated with initial dose of paroxetine (10 mg/kg/day), and no significant abnormal gross finding was confirmed by veterinarian. Therefore, we decreased the dosage to 5 mg/kg/day four and a half months following the initial treatment.

### Quantification of sertraline and paroxetine concentrations in serum and brain

To identify the sertraline and paroxetine concentrations in serum and brain, LC-MS/MS analysis, by using the mass spectrometer (Agilent triple quadrupole 6470), was performed on mouse samples after 12 months treatment of antidepressants based on the previous modified methods (Peng et al., 2008; Olesen et al., 2016). During sacrification, blood samples were collected by cardiac puncture and centrifuged for 10 min at 1,500 × rpm. The supernatant was stored at −80°C freezer for further analysis. 50 μL of serum was added with 5 μL of internal control (fluoxetine; concentration: 1,000 ng/mL), 50 μL sodium hydroxide (concentration: 0.1 M) and 0.5 mL ethyl acetate. The mixture was vortexed thoroughly for 2 min. The supernatant of the organic phase was collected and centrifuged at 13,000 × *g* for 5 min. For cerebellums, they were stored at −80°C after perfusion fixation by formaldehyde solution. Mouse cerebellums were added with 600 μL ethyl acetate and 5 μL of internal control (fluoxetine; concentration: 1,000 ng/mL), then homogenized by tissue homogeniser (Kurabo Sh-100) for 90 s at 1,600 × rpm, and then centrifuged at 2,500 × rpm for 10 min at 4°C. A volume of 300 μL top organic layer was further transferred to eppendorf tubes and centrifuged at 13,000 × rpm for 5 min. Both supernatant from processing of plasma and cerebellum was filtered by PVDF (0.22 μm) followed by LC-MS/MS analysis. Two MRM transitions were monitored for sertraline, paroxetine and fluoxetine (sertraline: 306.3–275.2; paroxetine: 330.1–192.2; fluoxetine: 310.1–148.1; Supplementary Figure S1). Calibration was performed by linear calibration based on nine-points and the range was 1–640 ng/mL.

### Quantification of serotonin and brain-derived neurotrophic factor (BDNF)

The serum samples were prepared as the same procedure described in the previous section for the quantification of sertraline and paroxetine. The serotonin and BDNF levels in the serum was quantified using the ELISA kits for ST/5-HT (5-hydroxytryptamine; FineTest EU0253) and for BDNF (Cloud clone SEA011Mu). Firstly, the plates were washed, and then 50 μL of standards, samples, or blanks were added to the pre-coated plate, along with 50 μL of biotin-labeled antibody. The plate was sealed, gently tapped, and incubated at 37°C for 45 min. Subsequently, a washing step was carried out, followed by the addition of 100 μL of HRP-Streptavidin Conjugate. After another washing step, 90 μL of 3,3′,5,5′-Tetramethylbenzidine (TMB) substrate was added, followed by 50 μL of Stop Solution. The absorbance of the plates at 450 nm was measured using the Bio-Tek µQuant Universal Microplate Spectrophotometer.

### Tissue preparation

The mice were sacrificed under a general anaesthesia overdose, followed by transcardial perfusion with ice-cold PBS and 10% formaldehyde. Subsequently, the brains were collected and kept in 10% formaldehyde for 24 h, followed by preservation in 70% ethanol at 4°C. The tissues were trimmed, and dehydrated with serial alcohol solution. They were then embedded in paraffin wax, and were cut to a thickness of 3 μm. Coronal brain tissue sections, intended for histopathological assessment, were obtained with the specific aim of capturing cuts that encompassed a sufficiently large area of the hippocampus. These sections were positioned approximately 2.3 mm caudal to the bregma, in accordance with the mouse brain atlas by Paxinos and Franklin (Paxinos and Franklin, 2001). This location corresponded to approximately 8.3 mm from the cranial apex.

### Hematoxylin and eosin (H&E)

For H&E staining, the paraffin section slides underwent deparaffinization with xylene and hydration using serial alcohol solutions. The sections were stained with hematoxylin solution (BioTnA TA01NB) for 1 min, followed by a 5-min wash with running tap water. Subsequently, the slides were counterstained with Eosin (BioTnA TA01ES) for 1 min. Dehydration with alcohol, clearance in xylene, and mounting with mounting medium followed.

Brain lesions in H&E-stained sections were assessed and graded by a veterinary histopathologist based on the INHAND (International Harmonization of Nomenclature and Diagnostic Criteria) (Shackelford et al., 2002). Evaluated areas and parameters included acidophilic material (characterized by strong eosinophilic deposits in the centre surrounded by pale-stained structures in a radial pattern), atrophy (decreased volume/cell counts), and gliosis (characterized by densely stained and small-sized elements) of the isocortex and hippocampus. Grading was performed as follows: Grade 0 - no remarkable histopathological changes (<1%); Grade 1 - minimal (1%–5%); Grade 2 - slight (5%–25%); Grade 3 - moderate (26%–50%); Grade 4 - moderately severe (51%–75%); Grade 5 - severe/high (>75%).

### Thioflavin-S staining

For thioflavin-S staining, the slides were deparrafinized and hydrated with xylene and serial alcohol solutions. Stains were performed with 1% thioflavin-S solution (Sigma-Aldrich) for 10 min, and then the slides were washed in running tap water for at least 10 min. The slides were then dehydrated with alcohol, cleared in xylene, and mounted with mounting medium.

Thioflavin-S staining was quantified both automatically, involving the calculation of the positive staining area relative to the entire brain, and manually, by counting the number of positive spots in the cortex and hippocampus. These assessments were conducted in a blinded fashion. Additionally, we engaged a veterinarian pathologist in identifying true positive signals to perform these assessments in a blinded manner.

### Congo red staining

Congo red staining was performed by using the stain kit (BIOTnA Biotech TASS12) to identify amyloids. After deparaffinization and hydration, the slides were stained by congo red solution for 15–20 min followed by rinsing in distilled water. They were then immersed in alkaline alcohol solution for differentiation and hematoxylin for counterstaining. Following dehydration, the slides were cleared in xylene and mounted with resinous mounting medium. We captured the images by using a compound optical microscope (Olympus, Japan) and analyzed the Congo red staining spots using ImageJ.

### Immunofluorescence and immunohistochemistry stainings

For immunofluorescent staining of anti-glial fibrillary protein (GFAP), following deparaffinization and hydration, the slides were blocked with 10% BSA in PBS and incubated with the primary antibody against GFAP (1:600; Genetex GTX108711) at 4°C overnight. Subsequently, the slides were incubated with the secondary antibody Alexa Fluor 488 (1:750; Abcam ab150077) for 1 hour at room temperature. Both primary and secondary antibodies were diluted in 3% BSA in PBS. The slides were mounted with a fluorescence mounting medium containing DAPI (Origene). Immunofluorescence was visualized and captured using a confocal microscope (Stellaris 8 confocal microscope, Leica).

For immunohistochemistry staining, the primary antibodies and their dilutions were anti-phospho-mixed lineage kinase domain-like protein Ser345 (pMLKL; 1:200; NovusBio NBP2-66953) and anti-ionised calcium-binding adapter molecule 1 (IBA1; 1:200; Bioworld BS90680). The slides were scanned using a MoticEasyScan Pro 6 at ×40 magnification.

For both staining techniques, positive-stained cells and total cell counts were manually calculated at ×40 magnification.

### Behavioural tests

To assess the long-term memory, mice were subjected to the novel object recognition test (NORT) based on modified methods from a previous study (Zhang et al., 2012). The novel object recognition tests were conducted at 3 and 12 months post antidepressants in a rectangular white arena (60 cm × 80 cm, surrounded by 60 cm-high walls). The entire assessment consisted of 4 phases: pre-habituation, habituation, training, and testing. On the first day, animals were brought to the testing room to freely explore the box in the absence of objects for 5 min. On the second and third day, mice were habituated to the empty box for 20 min per day. On the fourth day, each mouse took a training trial with exploration of two identical objects for 10 min and followed by a testing trial after an inter-session interval of 1 h. During the testing, animals were placed back to the same box, where one of the two familiar objects was switched to a novel one, to start a 5 min testing phase. Recording videos were analyzed by using ACTUAL TRACK software. Object exploration time was defined as the length of time the subject spent sniffing, pawing, or directed its nose within 2 cm of the object. Sitting or standing on the object was not recognized as exploration. The exploration time was analyzed manually using 2 stop watches. In the training session, the location preference in the training phase and recognition index (RI) in the testing phase were calculated using the following formula:

Recognition index (RI) = Time exploring novel object/(Time exploring novel object + Time exploring familiar object)×100%

The contextual fear conditioning was done at 15 months of age to evaluate by measurement of the tendency of freezing behavior. On training day, mice were given 3 min of exploration in a chamber with current-regulated shocker (Coulbourn Instruments) and followed by 2 s of electric footshock (0.75 Ma). Mice were then removed from the chamber 30 s later. The testing was performed 24 h following the training day, mice were returned to the chamber and freezing behaviour was recorded for 3 min. Freezing was defined as the absence of any movement with treshold of more than 1 s. Freezing was measured using the FreezeScan video tracking system and software (CleverSys).

The behavioral tests were carried out in a blinded manner, with the individuals conducting the tests unaware of the group assignments.

### Statistics

All of the data are presented as mean ± SD. All statistic analyses were performed using GraphPad Prism (GraphPad Software, Inc., San Diego, CA). The Log-rank test was used for survival analysis, and one-way ANOVA followed by Dunnett’s multiple comparisons test was employed for other comparisons. Significance was set at 0.05.

## Results

### Paroxetine increased body weight in both WT and APP/PSEN1 transgenic mice, while reduced survival in APP/PSEN1 mice

**FIGURE 1:**
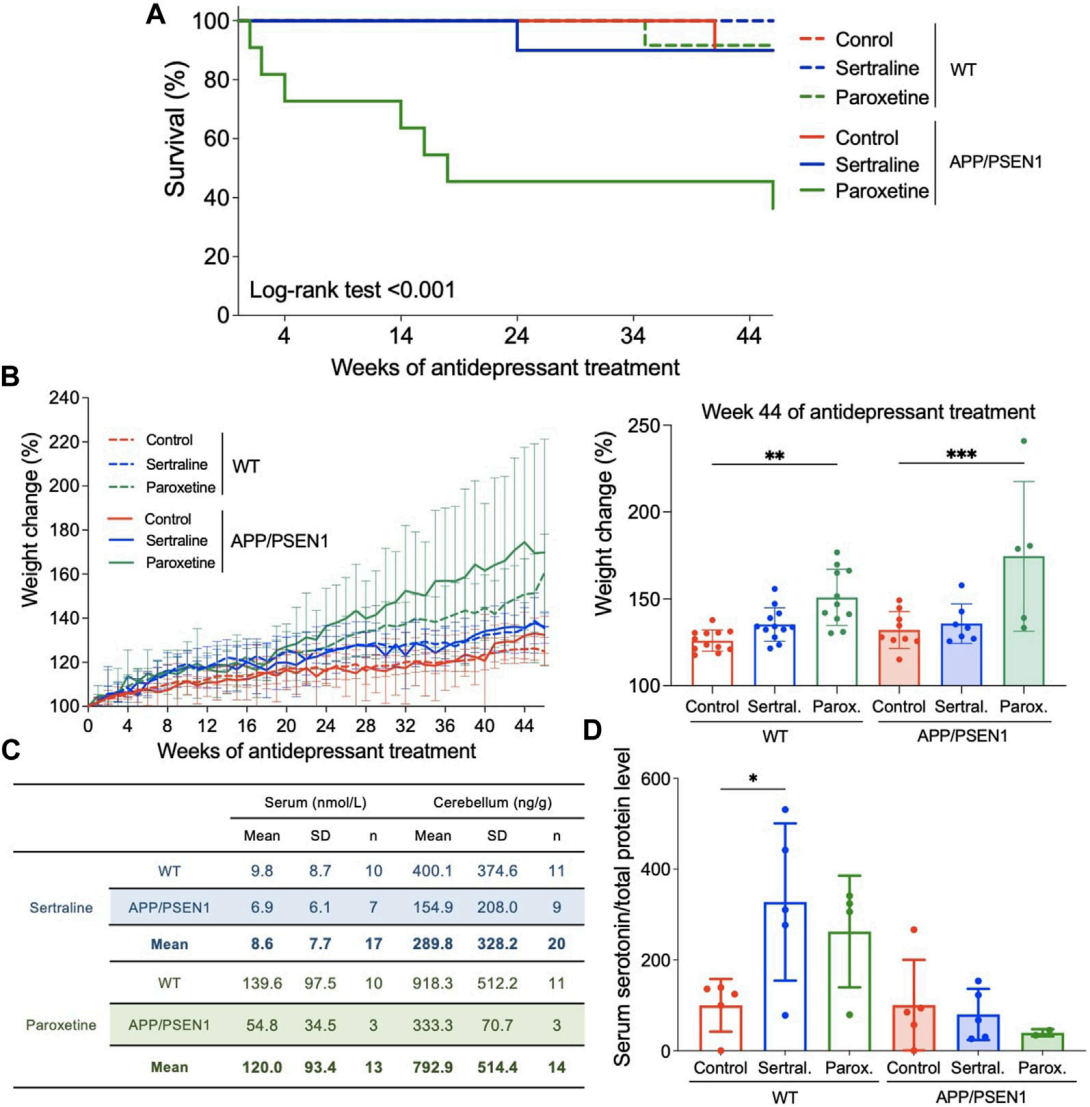
Sertraline and paroxetine increased body weight and accumulated to the level of hundreds ng/g in mouse cerebellum. **(A)** Kaplan-Meier (KM) plots of survival percentage among WT and APP/PSEN1 mice with different treatments. **(B)** Growth curve of mice in different groups (N = 60). Comparison of weight among different groups in WT and AD mice at week 44 after starting treatment treatment. **(C)** Sertraline and paroxetine levels in serum (nmol/L) and cerebellum (ng/g) tissues of WT and APP/PSEN1 mice fed with antidepressants for 12 months by using LC-MS/MS. **(D)** Serum serotonin levels of WT and APP/PSEN1 mice post antidepressant. Data are means ± SD. *p < 0.05, **p < 0.01, and ***p < 0.001.

Both WT and APP/PSEN1 mouse model were used to study the effect of antidepressants on initiation and progression of AD. Sertraline and paroxetine were administered in drinking water for a period of 12 months to study the long term effects of these medications. The daily dose of sertraline was 25 mg/kg/day, while paroxetine was initially given at a dosage of 10 mg/kg/day for the first four and a half months, and then reduced to 5 mg/kg/day for the remainder of the study. These initial dosages were chosen based on the recommendations provided in the guide for treating neuropsychiatric symptoms of AD patients (Lleo et al., 2006) and the dosages employed in animal models (Taler et al., 2013). Unexpected sudden mortalities happened in paroxetine group, which was limited to APP/PSEN1 mice (*p* < 0.001; Figure 1A). For both WT (*p* = 0.004) and AD (*p* < 0.001) mice, the groups treated with paroxetine demonstrated more weight gain compared to control groups (Figure 1B).

### High levels of sertraline and paroxetine were detected in cerebellums

To determine the antidepressant levels in systemic circulation and central nervous system, we performed LC-MS/MS analysis by using serum and cerebellum tissues. Data confirmed the existence of sertraline in our samples with acquisition time of 7.964 min and MRM transition of 306.3 to 275.2 (Supplementary Figure S1A), and acquisition time of 7.609 min and MRM transition of 330.0 to 192.0 for paroxetine (Supplementary Figure S1B). After pooling data from WT and APP/PSEN1 mice, sertraline was shown to be 8.6 nmol/L (n = 17) in serum and 289.8 ng/g (n = 20) in cerebellum, while paroxetine was 120.0 nmol/L (n = 13) in serum and 792.9 ng/g (n = 14) in cerebellum (Figure 1C). These results were in consistent with the serum concentrations of antidepressants from patients’ samples (Reis et al., 2009).

Sertraline and paroxetine belong to the class of SSRIs, which elevate extracellular serotonin levels by inhibiting its reabsorption by presynaptic cells. To determine whether the SSRI concentration was sufficient to impact the physiology of mice in this model, we quantified serum serotonin levels to assess the impact of the SSRI concentration on mouse physiology in this model. We observed a significant increase in serum serotonin levels in WT mice treated with sertraline (*p* = 0.01; Figure 1D), and a non-significant trend towards elevated serum serotonin levels in the paroxetine group. However, these effects were not observed in the APP/PSEN1 mice. As for BDNF, a protein crucial for stimulating the growth and preservation of neurons, no significant changes were observed among all groups (see Supplementary Figure S2).

### Sertraline significantly increased thioflavin-S and Congo red deposits in APP/PSEN1 mice

**FIGURE 2:**
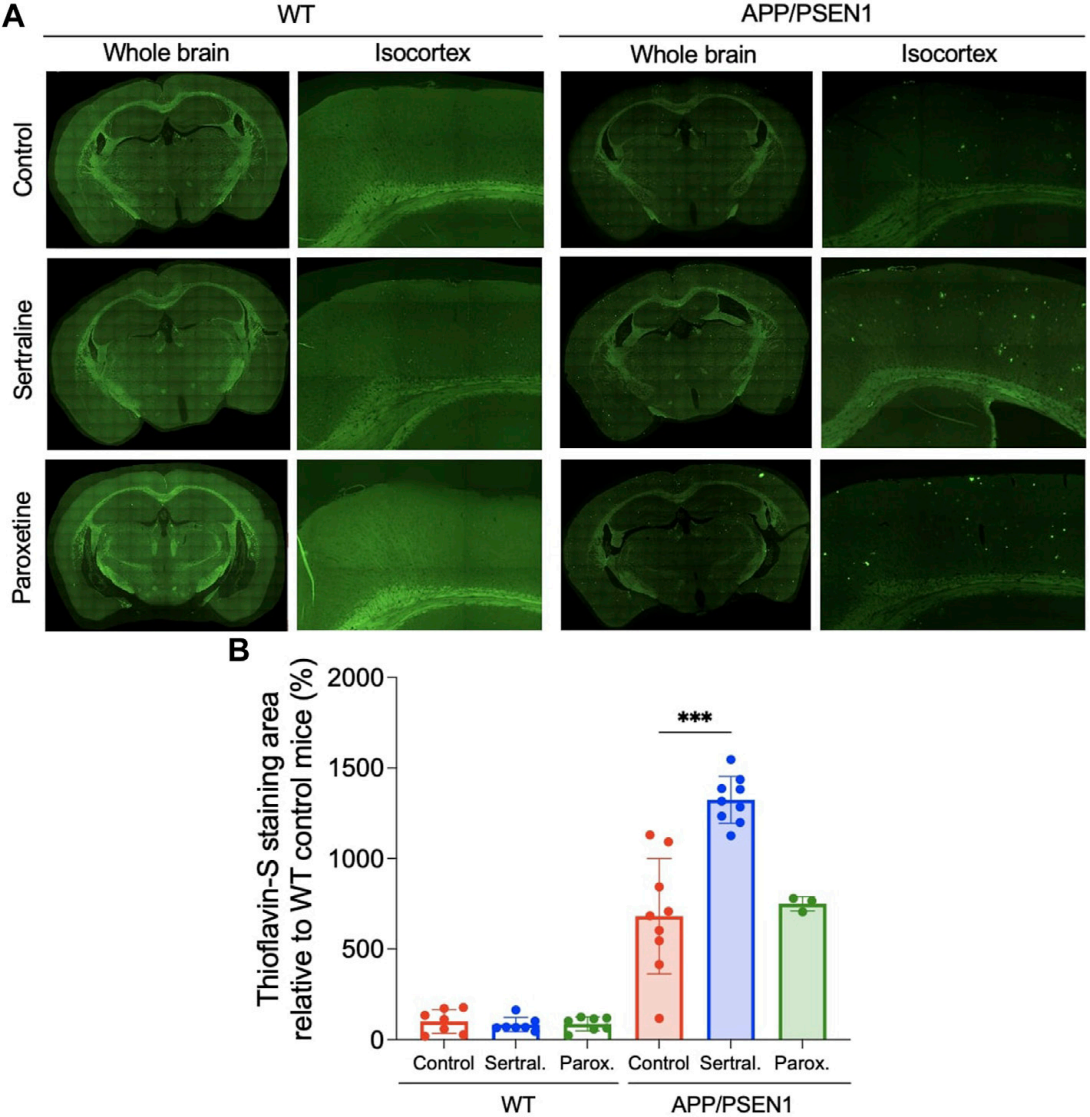
Sertraline increased thioflavin-S deposits in APP/PSEN1 mice. **(A)** Representative images of thioflavin-S staining in both WT and APP/PSEN1 mice. **(B)** Comparison of thioflavin-S deposition in WT and APP/PSEN1 transgenic mice after receiving only water (n = 9), sertraline (n = 9) and paroxetine (n = 3) for up to 12 months. Data are means ± SD. Comparison was done by ordinary one-way ANOVA followed by Dunnett’s multiple comparison test. *p < 0.05, **p < 0.01, and ***p < 0.001.

**FIGURE 3:**
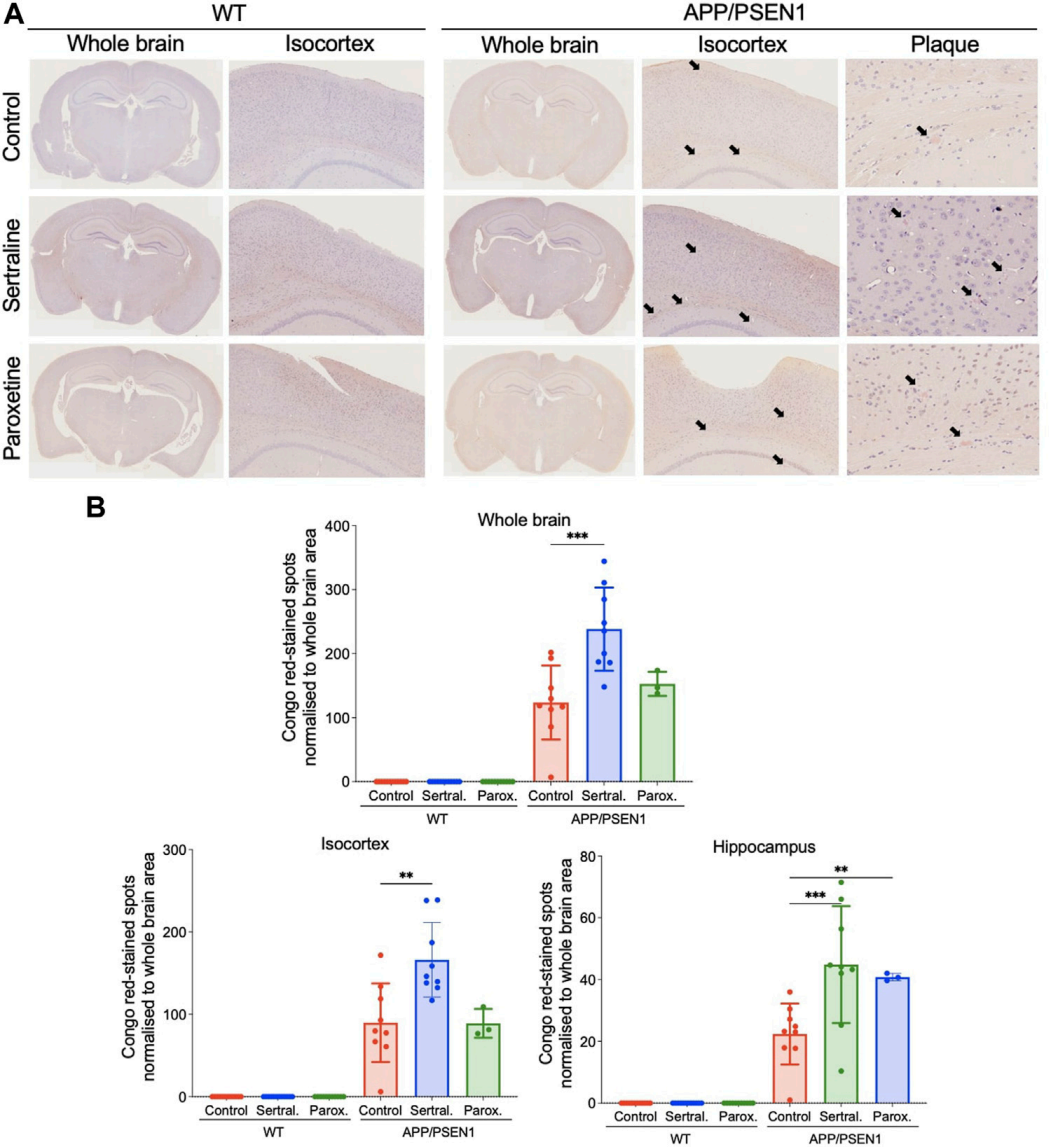
Sertraline increased Congo red-stained spots in APP/PSEN1 mice. **(A)** Representative images of congo red-stained spots in both WT and APP/PSEN1 mice (black arrows) and **(B)** the spot counts in the whole brain, isocortex and hippocampus. Data are means ± SD. Comparison was done by ordinary one-way ANOVA followed by Dunnett’s multiple comparison test. *p < 0.05, **p < 0.01, and ***p < 0.001.

By using thioflavin-S staining to evaluate the β-amyloid deposition, we observed minimal thioflavin-S deposit in WT mice across all treatment groups (Figure 2A left panel and Figure 2B). However, sertraline (*p* < 0.001) significantly increased the total area of thioflavin-S deposit compared to control group in APP/PSEN1 mice, as quantified automatically using ImageJ software (Figure 2A right panel and Figure 2B). This finding aligns with the result measured by a veterinary pathologist (*p* = 0.001; Supplementary Figure S3A). To further investigate the brain areas affected, we manually counted the thioflavin-S deposits in a blinded manner and observed a significantly higher number of deposits scattered across the isocortex (*p* < 0.001) and hippocampus (*p* < 0.001; Supplementary Figure S3B). These findings were supported by Congo red staining, which revealed that sertraline increased deposits in both the isocortex (*p* = 0.003) and hippocampus (*p* < 0.001) of APP/PSEN1 mice (Figure 3). We also found that paroxetine exacerbated the deposition in the hippocampus of APP/PSEN1 mice (*p* = 0.004).

### Sertraline induced elevated acidophilic material and gliosis in the isocortex and hippocampus of APP/PSEN1 mice

**FIGURE 4:**
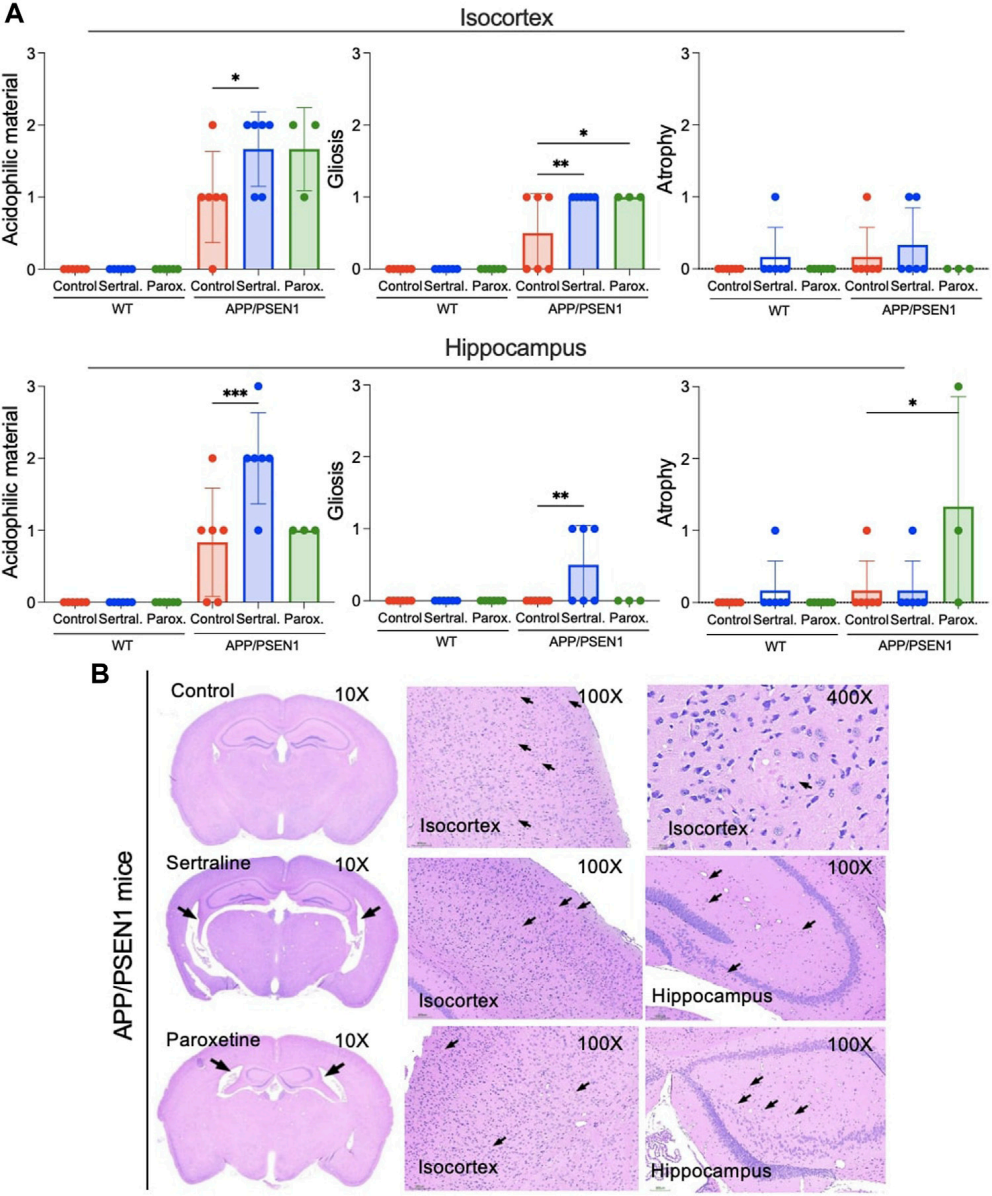
Sertraline induced an increase in the acidophilic material and gliosis in the isocortex and hippocampus in APP/PSEN1 mice. **(A)** Statistical analysis of severity of the acidophilic material, gliosis and atrophy in the isocortex and hippocampus of APP/PSEN1 mice graded by veterinary histopathologist based on the INHAND. **(B)** APP/PSEN1 mice were observed with only a mild level of the acidophilic material in the control group. It was significantly higher in the sertraline group. Arrows indicate the brain lesions. Data are means ± SD. Comparison was done by ordinary one-way ANOVA followed by Dunnett’s multiple comparison test. *p < 0.05, **p < 0.01, and ***p < 0.001.

In the APP/PSEN1 mice, elevated levels of acidophilic material and gliosis were observed in both the isocortex (*p* = 0.020 and = 0.004) and hippocampus (*p* < 0.001 and = 0.004) of the sertraline-treated group when compared to the control group (Figures 4A, B). It is noteworthy that the acidophilic materials were surrounded by glial cells (Supplementary Figure S4). Additionally, there was a non-significant trend indicating an increased presence of Iba-1^+^ cells, a microglial marker, in the sertraline-treated APP/PSEN1 mice (Supplementary Figure S5). In contrast, paroxetine significantly exacerbated the gliosis (*p* = 0.020) and also led to hippocampal atrophy (*p* = 0.010) in the APP/PSEN1 mice (Figure 4A, middle and right panels). This treatment was also associated with a trend of increased GFAP^+^ cells, an astrocyte marker, in both WT and APP/PSEN1 mice (Supplementary Figure S5).

### A trend of decline of short-term memory in sertraline and paroxetine groups of APP/PSEN1 mice at 12 months post antidepressants

**FIGURE 5:**
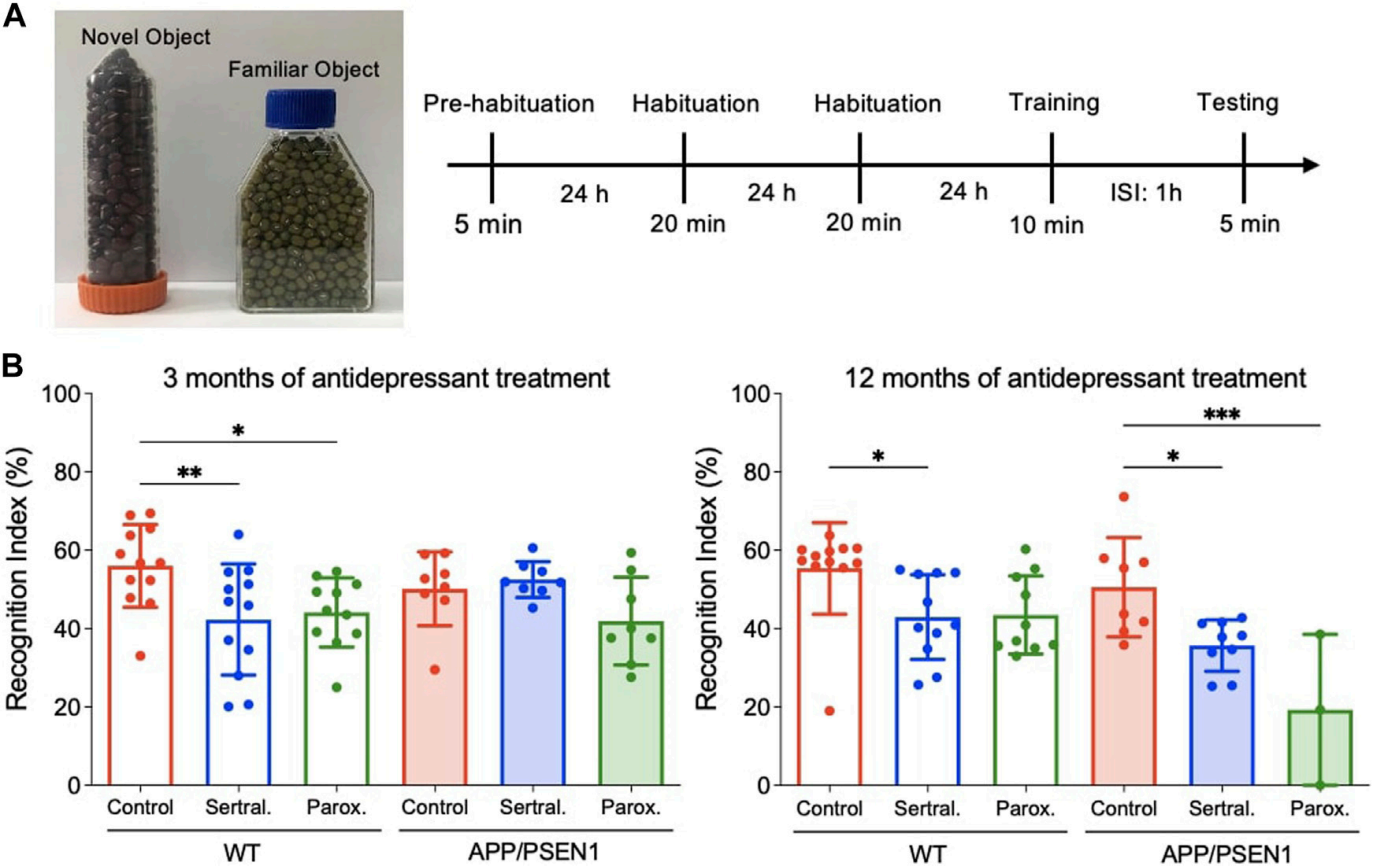
Sertraline and paroxetine worsened short-term memory in 15-month-old APP/PSEN1 mice. **(A)** Schematic diagram of novel object recognition test. **(B)** Recognition index of different groups at 6 month-old and 15-month old. Data are means ± SD. Comparison was done by ordinary one-way ANOVA followed by Dunnett’s multiple comparison test. *p < 0.05, **p < 0.01, and ***p < 0.001.

Novel object recognition test was performed to evaluate the cognitive function of mice with or without antidepressants (Figure 5A). The results showed sertraline (*p* = 0.009) and paroxetine (*p* = 0.04) significantly reduced recognition memory of WT mice 3 months after antidepressants administration (Figure 5B left), while the cognition of APP/PSEN1 mice in sertraline (*p* = 0.03) and paroxetine (*p* < 0.001) group were decreased in 12 months (Figure 5B right). However, no significant difference among groups was found in the contextual fear conditioning (Supplementary Figure S6). The result implied that sertraline and paroxetine impair memory function after long-term antidepressant medication.

## Discussion

This is the first study to demonstrate that sertraline and paroxetine enhanced the thioflavin-S deposition in APP/PSEN1 mice, indicating these two SSRIs might accelerate the pathogenesis of AD. Thioflavin-S deposit was not detected in WT mice, regardless of control or antidepressant groups, which suggests that antidepressant alone is not sufficient to induce the formation of β-amyloid. The chronic toxicity of paroxetine was observed exclusively in APP/PSEN1 mice, emphasizing the importance of studying the potential side effects of this medication in AD patients.

*In vitro* studies have shown that sertraline and paroxetine, below 10 μM, promote neurogenesis and ameliorate inflammatory response (Anacker et al., 2011; Peng et al., 2013; Lu et al., 2019). In contrast, above 10 μM, sertraline and paroxetine are toxic to astrocytes and neurons (Then et al., 2017b). It is worth investigating the effects of the cumulative concentration of antidepressants on the central nervous system in mouse models and human studies because of these seemingly conflicting results. In order to study the concentration which is achievable in patient in clinical settings, we used the dosage prescribed for patients (Lleo et al., 2006) in this study. We performed high performance liquid chromatography to determine the concentration of sertraline and paroxetine in brain and serum. Our results aligns with the serum concentration of 131 nmol/L for paroxetine, as proposed by Reis et al., 2009. However, in contrast to their study, our findings indicated a lower level of paroxetine compared to the reported concentration of 67 nmol/L. In addition, Peng et al. showed that the brain/blood ratio of sertraline is more than 45 which showed a high level of antidepressant in the brain tissue compared to serum (Peng et al., 2008). We also found high levels of sertraline and paroxetine in brain tissue which suggests the increased thioflavin-S and Congo red deposits could be the consequence of exposure to high level of antidepressant medication.

To assess the cognitive function of mice, we conducted novel object recognition tests at 3 months and 12 months post-antidepressant administration. At 3 months, impaired recognition memory was observed in the sertraline and paroxetine groups of WT mice. It is essential to note that control AD mice already exhibited a tendency towards a lower recognition index compared to control WT mice at this time point, and no significant differences in the recognition index were observed among all groups of APP/PSEN1 mice. At 12 months, in addition to the impaired recognition function observed in sertraline-treated WT mice and a non-significant trend in the paroxetine group, we also found a decline in the recognition index in the antidepressant groups of APP/PSEN1 mice. This suggests that memory was impaired by sertraline and paroxetine in aged AD mice, leading them to spend less time exploring a novel object. Sperling et al. suggested that the deterioration of clinical function may occur at a later stage, subsequent to the presence of elevated levels of β-amyloid and cognitive impairment (Sperling et al., 2011). We found that the weight of mice treated with sertraline and paroxetine were significantly higher than that of the control group. This result suggested that the antidepressants, given for a duration of 12 months, may decrease mouse metabolism. We also conducted a contextual fear conditioning test and found no significant differences among the groups. Although both behavior tests evaluate memory and learning capabilities, they differ in the type of memory they assess and the brain regions involved. The novel object recognition test focuses on non-associative memory and engages brain regions linked to the medial temporal lobe, including the hippocampus and perirhinal cortex (Kinnavane et al., 2016). In contrast, contextual fear conditioning examines associative, emotional, and fear-related memory and also relies on the hippocampus for context encoding (Kim and Cho, 2020). Furthermore, it is noteworthy that pathological changes consistently preceded observable behavioral changes (Sperling et al., 2011). In this model, β-amyloid deposits develop by 6 months, and cognitive deficits become detectable at 12 months (Janus et al., 2015). It is possible that the memory deficits observed may not be severe enough to manifest in both tests.

To further study the possible mechanism of sertraline-increased thioflavin-S and Congo red deposits, we further investigated other histological phenotypes in these mice. In APP/PSEN1 mice treated with sertraline, we observed mild gliosis in the isocortex and hippocampus, surrounding the acidophilic material. However, the causal relationship between thioflavin-S and Congo red deposition and gliosis remains elusive in this study, as gliosis could be either the cause or consequence of β-amyloid accumulation. Previous studies have shown controversial results of pro- and anti-inflammatory effects of sertraline and paroxetine (Maes et al., 1999; Marques-Deak et al., 2007; Tynan et al., 2012), while inflammation has been proposed as a central mechanism in exacerbating the pathogenesis of AD (Kinney et al., 2018). Notably, an *in vitro* study has demonstrated that both sertraline and paroxetine elevate BDNF levels and offer neuroprotection by enhancing the overall outgrowth of hippocampal dendrites under toxic conditions. (Seo et al., 2014). In addition, sertraline also reduced inflammatory processes in the rat hippocampus during the onset of seizures (Sitges et al., 2014). Moreover, sertraline and paroxetine suppressed microglial responses to an inflammatory stimulus by reducing tumour necrosis factor-alpha (TNF-alpha) and nitric oxide (NO) production after stimulation with lipopolysaccharide (Tynan et al., 2012). A more in-depth investigation into the relationship between AD pathology and neuroinflammation, including the involved immune cells and biological processes, is warranted.

In this section, we review other studies and discuss the impact of chronic SSRI treatment on the formation of β-amyloid, considering potential outcomes such as protective effects, no discernible impact, or an increase in plaque load. Consistent with our results, Severino et al. and Sivasaravanaparan et al. showed that chronic paroxetine therapy, administered at a dosage of 10 mg/kg/day in drinking water, elevated mortality rates in APP/PSEN1 transgenic mice aged 9–18 months (Severino et al., 2018; Sivasaravanaparan et al., 2022). Notably, a substantial improvement in survival outcomes was observed when the dosage was reduced to 5 mg/kg/day during the same age range. It is worth noting that paroxetine did not exhibit a reduction in amyloid pathology in the neocortex and hippocampus of APP/PSEN1 transgenic mice, as assessed through immunohistochemistry staining of β-amyloid. Additionally, the administration of paroxetine led to an increase in body weight in WT mice. Our investigation on APP/PSEN1 mice demonstrated that paroxetine also increased mortality, as well as Congo red-stained deposition in the hippocampus. In a 3xTg AD mouse model, daily intraperitoneal injections of 5 mg/kg paroxetine over 5 months significantly decreased levels of amyloid β-peptide in hippocampal and neocortical tissues, as determined by ELISA assay (Nelson et al., 2007). Additionally, the number of β-amyloid immunoreactive neurons in the hippocampus reduced, as assessed using IHC. While Tin et al. showed a 70% inhibition of β-amyloid aggregation at 100 μM of paroxetine in the aggregation kinetic experiment, this finding has not been validated in cell or animal studies (Tin et al., 2018). Our *in vitro* study found that this concentration is five times higher than what could induce astrocyte apoptosis (Then et al., 2017b).

Amoxapin, a tricyclic antidepressant (TCA), at a concentration of 10 μM for 24 h, has been reported to reduce β-amyloid generation as assessed using ELISA analysis in a human neuroblastoma cell line (Li et al., 2017). This reduction involves multiple serotonin receptor 6 (HTR6)-mediated targets, including β-arrestin2 and CDK5 (Li et al., 2017). This aligns with evidence indicating that TCAs are relatively safe for astrocytes compared to sertraline and paroxetine (Then et al., 2017b). Furthermore, Sheline et al. demonstrated that intraperitoneal citalopram, found to be non-cytotoxic to astrocytes in our *in vitro* study, reduced Aβ levels in brain interstitial fluid (ISF) (Sheline et al., 2014). This effect, observed using *in vivo* microdialysis probes implanted in the hippocampus, occurred in a dose-dependent manner, with doses ranging from 5 to 20 mg/kg administered intraperitoneally within 1 day in aged APP/PSEN1 plaque-bearing mice. Additionally, Cirrito et al. showed that other SSRIs, namely, fluoxetine (10 mg/kg), desvenlafaxine (30 mg/kg), and citalopram (5 and 10 mg/kg) administered intraperitoneally, reduced brain interstitial fluid (ISF) Aβ levels within 24 h in a mouse model of AD, as measured by *in vivo* microdialysis. Morever, a history of taking antidepressants within the past 5 years was associated with fewer cortical amyloid plaques in human participants, as quantified by PET imaging (Cirrito et al., 2011). Despite evidence supporting short-term treatments of antidepressants from different classes, such as amoxapine (TCA) and citalopram (SSRI) in mitigating Aβ deposition, there is a lack of studies concerning chronic administration of sertraline and paroxetine. To our knowledge, this study is the first to show long-term usage of sertraline increases thioflavin-S and Congo red deposition in APP/PSEN1 mice, along with a decline in recognition function.

## Conclusion

In this study, sertraline accumulated in brain tissues and significantly increased thioflavin-S and Congo red deposits in the isocortex and hippocampus of APP/PSEN1 mice. Additionally, these antidepressants were found to enhance gliosis and impair cognitive function. Further research is necessary to investigate the safety of antidepressant medication, specifically sertraline and paroxetine, when used by patients with AD.

## Data Availability

The original contributions presented in the study are included in the article/Supplementary Material, further inquiries can be directed to the corresponding authors.
