# Supplementary material for: Antidepressant sertraline increases thioflavin-S and Congo red deposition in APPswe/PSEN1dE9 transgenic mice
Source: Front Pharmacol. 2024 Jan 8;14:1260838. doi: 10.3389/fphar.2023.1260838 (PMC10800414; doi:10.3389/fphar.2023.1260838)
Supplement: Supplementary file 1 [file Presentation1.pdf]

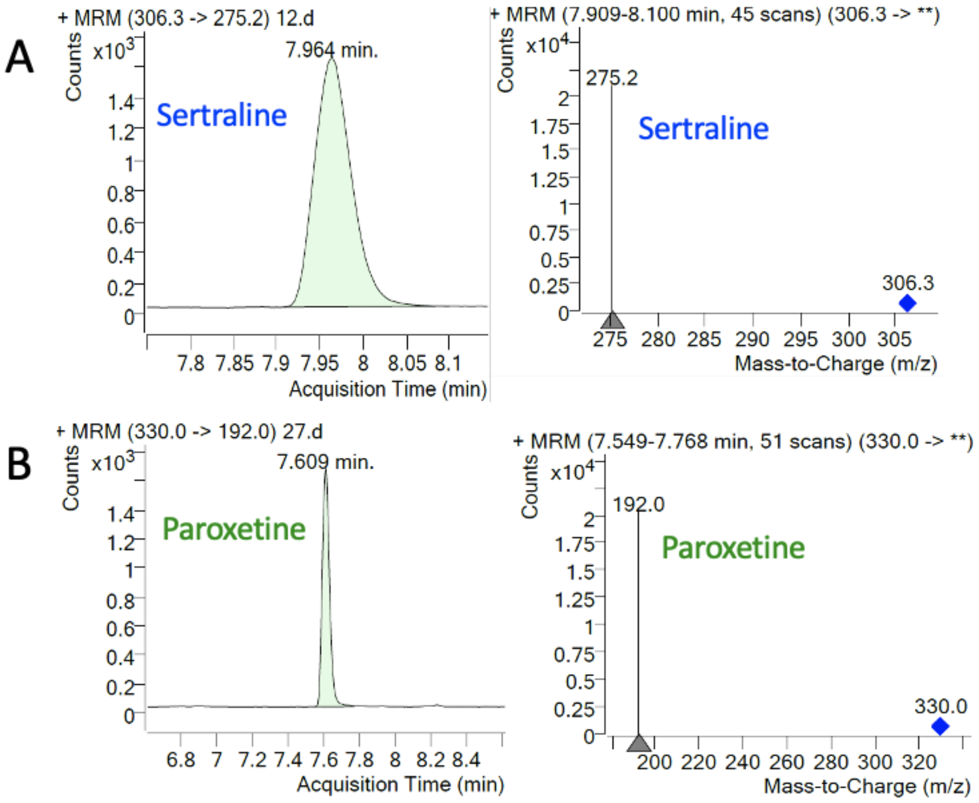

Supplementary Figure 1 LC-MS/MS analysis of sertraline and paroxetine by using serum and cerebellar tissues.

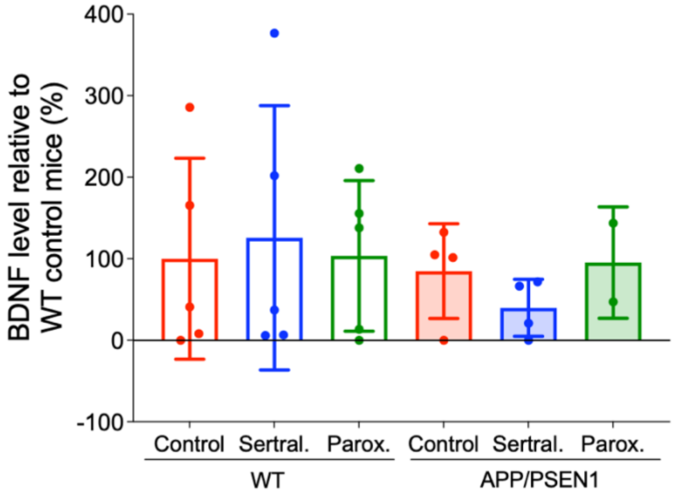

Supplementary Figure 2 Serum BDNF levels in WT (n=5/group) **and APP/PSEN1** (n=4 for control and sertraline, n=2 for paroxetine) **mice following antidepressant treatment.** Data are means ± SD. Comparison was done by ordinary one-way ANOVA followed by Dunnett's multiple comparison test.

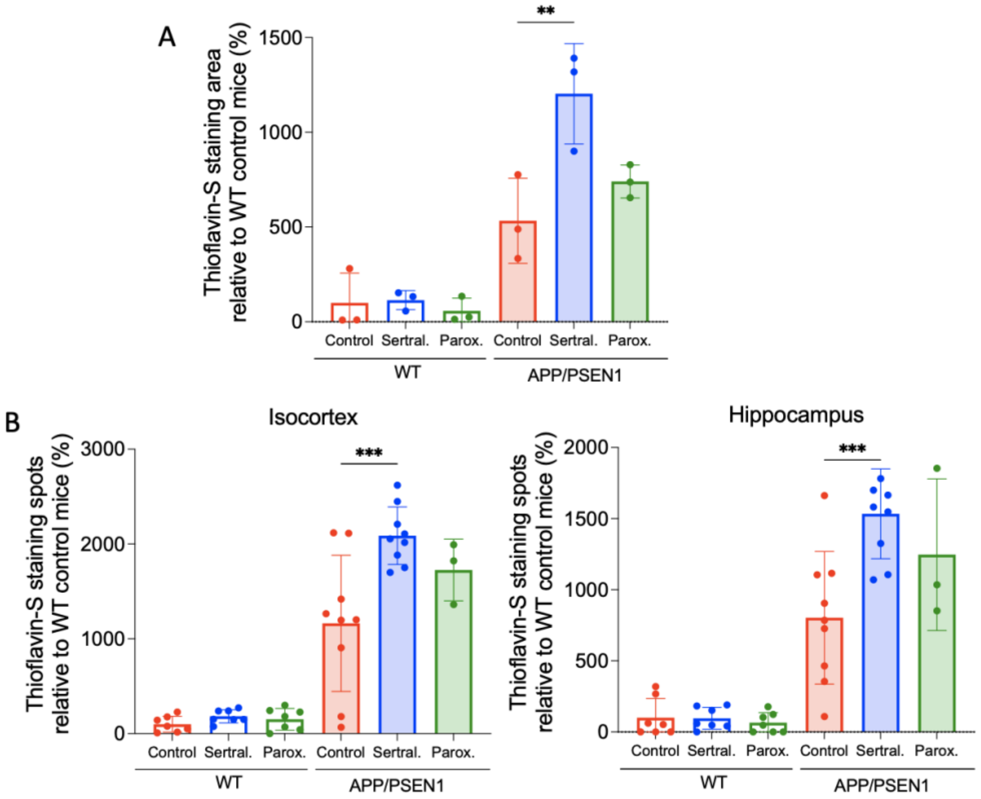

Supplementary Figure 3 Sertraline increased thioflavin-S deposits in cortex and hippocampus of APP/PSEN1 mice.

(A) Consistent findings reveal a marked increase in thioflavin-S$^+$ deposits in the sertraline-treated group compared to the control group in APP/PSEN1 mice. This assessment was performed through an automated, blinded analysis by a veterinary histopathologist on WT (n=3/group) and APP/PSEN1 (n=3/group) mice. (B) A comparison of the number of thioflavin-S$^+$ spots in the isocortex and hippocampus of both WT (n=7/group) and APP/PSEN1 (n=9 for control and sertraline, n=3 for paroxetine) mice, using a manual, blinded assessment. Data are means ± SD. Comparison was done by ordinary one-way ANOVA followed by Dunnett's multiple comparison test. *P<0.05, **P<0.01, and ***P<0.001.

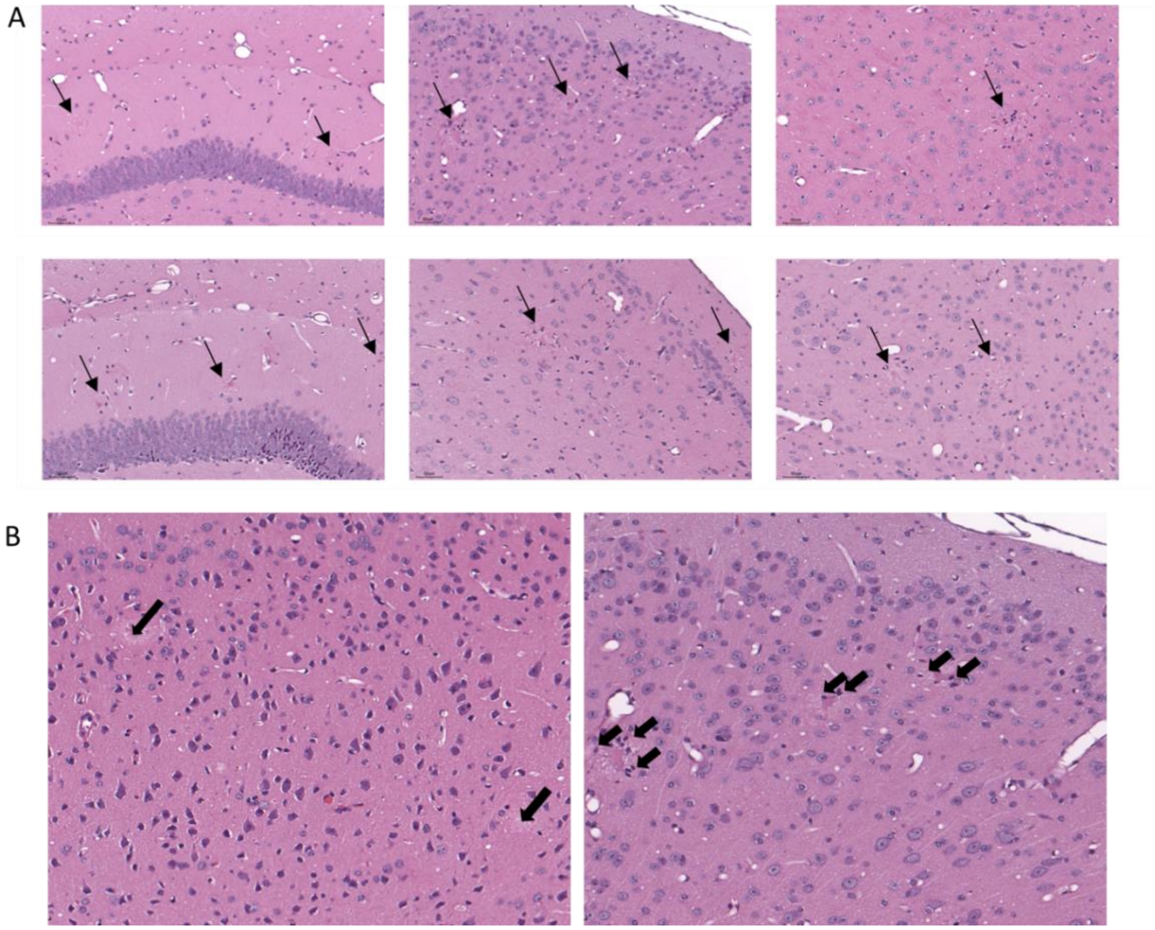

Supplementary Figure 4 Sertraline increased acidophilic material, along with gliosis, in APP/PSEN1 mice.

(A) Representative images of acidophilic material (black arrows) in the control (upper panel) and sertraline (lower panel) groups of APP/PSEN1 mice. The morphology mainly consists of two parts: irregular, intensely eosinophilic-stained deposits in the center, and faintly stained structures with irregular or radial patterns in the periphery. (B) Representative images of gliosis surrounding the acidophilic material. Glial cells (black arrows), characterized by densely stained and smaller size compared to neurons, aggregate around the acidophilic material.

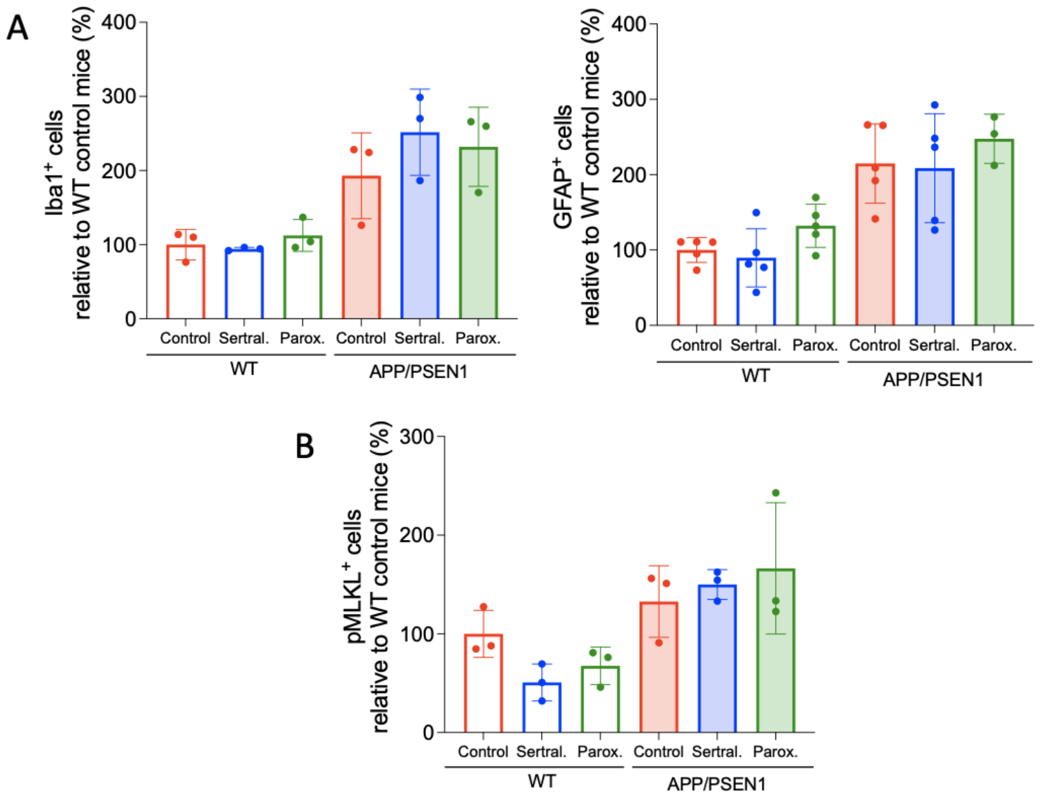

Supplementary Figure 5 Cells positively stained for Iba1, GFAP, and pMLKL in WT and APP/PSEN1 mice.

Quantitative analysis of (A) Iba1 (n=3/group in WT and APP/PSEN1) and GFAP (n=5/group in WT; n=5 for WT, n=5 for sertraline and n=3 for paroxetine in APP/PSEN1), marking microglial cells and astrocytes, and (B) pMLKL (n=3/group in WT and APP/PSEN1), indicative of necrosis, through immunohistochemistry staining. Data are presented as means ± SD. Statistical comparison was conducted using ordinary one-way ANOVA followed by Dunnett's multiple comparison test.

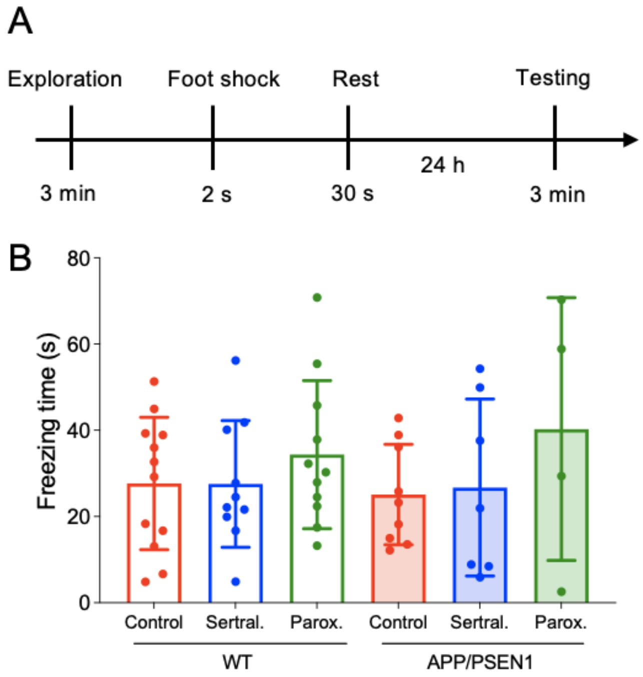

Supplementary Figure 6 Contextual fear conditioning
(A) Schematic diagram illustrating contextual fear conditioning of WT (n=12 for control, n=10 for sertraline, n=11 for paroxetine) and APP/PSEN1 (n=9 for control, n=7 fpr sertraline, n=4 for paroxetine) mice. (B) Freezing time of various groups at 15 months old. Data are presented as means ± SD. Statistical comparison was performed using ordinary one-way ANOVA followed by Dunnett’s multiple comparison test.
